# Chemical Profiling and Antioxidant Activity of *Tanacetum vulgare* L. Wild-Growing in Latvia

**DOI:** 10.3390/plants12101968

**Published:** 2023-05-12

**Authors:** Renāte Šukele, Liga Lauberte, Liga Kovalcuka, Konstantins Logviss, Ance Bārzdiņa, Agnese Brangule, Zoltán Márk Horváth, Dace Bandere

**Affiliations:** 1 Department of Pharmaceutical Chemistry, Riga Stradiņš University, LV-1007 Riga, Latvia; 2 Department of Pharmaceuticals, Red Cross Medical College of Riga Stradiņš University, LV-1009 Riga, Latvia; 3 Laboratory of Finished Dosage Forms, Riga Stradiņš University, LV-1007 Riga, Latvia; 4 Clinical Institute, Faculty of Veterinary Medicine, Latvia University of Life Sciences and Technologies, LV-3004 Jelgava, Latvia; 5 Baltic Biomaterials Centre of Excellence, Headquarters at Riga Technical University, LV-1658 Riga, Latvia

**Keywords:** *Tanacetum vulgare*, aerial part, phenolic compounds, thujone, antioxidant activity

## Abstract

The *Tanacetum vulgare* L. (Tansy) has several ethnobotanical uses, mostly related to the essential oil and sesquiterpene lactones, whereas information regarding other compounds is scarce. This research is designed to characterize the phenolic compounds (flavonoids, phenolic acids, and tannins) to analyze the thujone (which is toxic in high concentrations) content and to detect the antioxidant activity (DPPH assay) of extracts. The main highlights of our work provide a chemical profile of phenolic compounds of *T. vulgare* harvested from different regions of Latvia, as well as simultaneously support the ethnomedicinal uses for wild *T. vulgare* through the integration of phenolic compounds as one of the value constituents of leaves and flowers. The extraction yield was 18 to 20% for leaves and 8 to 16% for flowers. The total phenol content in the extracts of *T. vulgare* as well as their antioxidant activity was different between collection regions and the aerial parts ranging from 134 to 218 mg GAE/g and 32 to 182 mg L^−1^, respectively. A remarkable variation in the thujone (α + β) content (0.4% up to 6%) was detected in the extracts. *T. vulgare* leaf extracts were rich in tannins (up to 19%). According to the parameters detected, the extracts of *T. vulgare* could be considered promising for the development of new herbal products.

## 1. Introduction

*Tanacetum vulgare* L., commonly known as tansy, is an intensely aromatic plant of the Asteraceae family, native to Europe and Asia, where it grows along roadsides, hedges, and wastelands [1]. *T. vulgare* subspecies *vulgare* is a common plant in Latvia, and its range of distribution covers the whole country [2]. It has traditionally been used as a spicy additive for food, in cosmetics, and as a herbal remedy due to its biologically active compounds. Besides the scientifically important and closely related species, feverfew (*T. parthenium*) and dalmatian insect flower (*T. cinariifolium*), significant applications for common tansy have not yet been found [3].

*T. vulgare* has been used for centuries as a medicinal plant and flavoring herb, but in order to confirm its therapeutic value, detailed phytochemical and pharmacological analyses are still required [4,5].

Plant extracts and essential oil of *T. vulgare* are known for their biological activities, mainly antioxidant, antimicrobial, insecticidal, cytotoxic, antivirus, and anti-inflammatory activities [6,7]. The extracts of *T. vulgare* have been reported to have anticancer properties and an antiproliferative effect on human cervical adenocarcinoma (HeLa) cells in the micrograms range [6,8]. However, pharmaceutical activities are mainly attributable to specific compounds, such as sesquiterpene lactones, volatile oil, flavonoids, and phenolic acids [4,9,10]. The evaluation of the approximate composition of *T. vulgare* in scientific research focuses primarily on determining the composition and biological properties of essential oil, while information on other compounds is scarce [1,5,11,12,13].

Chemovariation is a well-known fact in *Tanacetum* species, which is encountered at the species level and at the subspecies level. Essential oil isolated from *Tanacetum* species has variable chemical constituents and is made of more than 200 compounds [14]. The non-volatile extractives of *T. vulgare* are mainly characterized as hydrophilic antioxidants [12]. So far, neither the basic composition of the leaves nor the tansy inflorescences have been analyzed. *T. vulgare* is considered a rich source of sterols (stigmasterol, campesterol, and cholesterol), non-volatile sesquiterpene lactones (tanacetine, parthenolide, and tanachine), flavonoids (including luteolin, quercetin, apigenin, and their glycosides), and phenolic acids (chlorogenic, caffeic, and dicaffeoylquinic acids) [9,15,16]. The high variation of secondary metabolites in *T. vulgare* still appears to be a problem for further research as well as the optimization and standardization of cultivation conditions.

The use of extracts and essential oil of *T. vulgare* is limited by the presence of toxic thujones [1,17]. Thujones are bioactive compounds with valuable medicinal properties; however, they are toxic at high concentrations [18,19]. Certain amounts of thujone are allowed in foods and other products in the European Union. Due to the toxic effects of thujones, tansy flower extracts are mainly used externally as antiparasitic agents [1].

The biological and pharmacological assays are reported to strongly depend not only on the morphological part of *T. vulgare* but also on the extraction solvents used [4,18,20]. Generally, hydroalcoholic extracts and infusions could be considered more active compared to lipophilic n-hexane extracts. In our investigation, we obtained hydroalcoholic extracts directly without any previous separation of lipophilic extractives. In all experiments, the crude extracts were used without any modification. An important advantage of using extracts versus isolated molecules is the presence of other molecules in the extract that can synergistically interact with the bioactive compound, potentiating its beneficial effect [21].

Identifying bioactive compounds is the initial step for drug discovery. Target-based screening is used mainly to identify compounds that modulate the activity. Firstly, the assays can be selected considering that structurally similar compounds have similar biological activity. However, it must be noted that similarity in structure does not always mean similar biological activity, since even a single change in a chiral center can cause significant changes in activity (e.g., the Thalidomide tragedy) [22]. The investigation of antioxidant properties is mandatory in almost every study of biological activities [21]. This is because oxidant stress caused by an excessive amount of oxidants is associated with a number of human ailments, which include but are not limited to cancer and the development of neurological disorders and cardiovascular diseases [23]. Our research is designed to identify the major chemical active groups, focusing on phenolic compounds as one of the most abundant phytochemicals with antioxidant properties.

The aim of this study was to determine and compare the chemical composition of the phenolic compounds and the thujone content, as well as the antioxidant activity of aqueous ethanol (50%, *v*/*v*) extracts of *T. vulgare* wild-growing in different regions of Latvia.

## 2. Results

### 2.1. Extraction Yield of T. vulgare

The dryness of the analyzed leaves, stems, and aerial parts of *T. vulgare* was even and averaged at 5.5%. The exception was tansy flowers with detected moisture up to 9%. Considering that tansy flowers are rich in essential oil and that the gravimetric method was applied, the mass loss could be attributed not only to water content but also to essential oil content.

**Figure 1:**
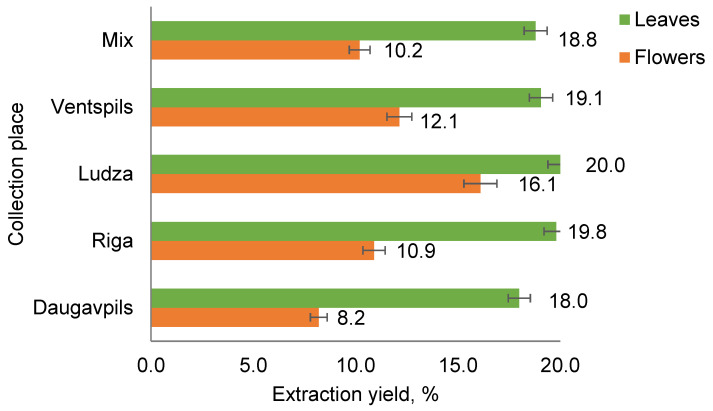
Extraction yields of *T. vulgare* flowers and leaves collected from different places.

Depending on the collection place, the leaf extracts had the highest extraction yields of 18 to 20% (*w*/*w*) per dry biomass of *T. vulgare*. The biomass samples of *T. vulgare* obtained by mixing an equal proportion of leaves or flowers together without sorting them according to their collection place were designated as mix samples. The flower extracts had greater variation in extract yields (8 to 16%, *w*/*w*), depending on the collection place (Figure 1), which could be explained by the higher content of volatile substances, mainly essential oil in the samples. This chemical divergence concerns both major and minor compounds.

In order to evaluate the extraction yield of different morphological parts of wild *T. vulgare*, the sample harvested in Daugavpils was additionally separated into leaf, flower, and stem parts (Table 1).

The highest 50% aqueous ethanol extraction yield of *T. vulgare* was found in the leaves (18%), followed by the aerial part (14%), stems (11%), and flowers (8%).

### 2.2. Phenolic Profile of T. vulgare Extracts

Polyphenols are broad and diverse plant secondary metabolite groups. Their structures range from quite simple molecules, such as phenolic acids, to complex polymerized molecules such as tannins [24]. Furthermore, they appear in conjunction with various sugars in plants. Due to the chemical diversity of phenolic compounds and the complexity of the composition in plant samples, it would be costly and inefficient to separate each phenolic compound and study them individually [25]. However, it is important to identify the main groups and then characterize the types of phenolic compounds [26].

An important group of secondary metabolites presented in *T. vulgare* is phenolic compounds [9].

Weather conditions can influence the chemical composition and amount of compounds in the plant. The year 2022 was extremely warm, especially in summer when the average temperatures were 0.5 °C above the norm of about 19.8 °C, making it the hottest year since 1924. The temperature of herb harvest sites differed from each other. The average temperature was higher in Ventspils, Daugavpils, Ludza, and Riga by 2.6 °C, 2.9 °C, 3.0 °C, and 3.1 °C, respectively. Even though at the end of August, the average temperature dropped by 11.3 °C, it was still 6.4 °C above the norm. Furthermore, insolation time was longer than average by 15% over the norm. The precipitation was 3% (222.6 mm) over the norm; however, most raindrops were observed in the northern central parts of Latvia (Zoseni), while the least was in the central and southern parts of Latvia. Cases of extreme rainfall were observed throughout all of the study regions during the summer with periods of drought. During summer, August had the least precipitation. In August, out of all the places that were studied, the least amount of precipitation was observed in Riga, and the highest amount of rainfall was observed in Ventspils.

The results in Table 2 indicate that *T. vulgare* growing at different sites in Latvia is rich in phenolic compounds. The total phenolic content ranges from 127 to 155 mg GAE/g in the flower extracts and from 135 to 219 mg GAE/g in the leaf extracts. Furthermore, depending on the harvest location, all extracts contained at least 15 mg QE/g of flavonoids and 11 mg CAF/g of phenolic acids. The highest total flavonoid content (28 mg QE/g) was observed in the leaf extract of *T. vulgare* harvested in Ludza. The most abundant in phenolic acids (31 mg CAF/g) was the leaf extract of T. vulgare harvested in Daugavpils.

The data from the literature show that the highest content of total phenols are in the extracts of *T. vulgare* leaf, followed by extracts of flower and stem [6,27]. The results in Table 3 are in good agreement with these findings. The highest total phenolic content was observed in the leaf extract (156 mg GAE/g) and then in the flower extract (134 mg GAE/g) and in the stem extract (127 mg GAE/g). However, the highest amount of phenolic compounds (240 mg GAE/g), including flavonoids (85 mg QE/g), was obtained from the aerial part of *T. vulgare*. The highest total phenolic acid content was in the leaf extract (31 mg CAF/g), and the lowest was in the flower extract (15 mg CAF/g).

Among the studied plant tissues (Table 3), leaves were the richest in phenolic compounds with 28.0 mg GAE/g of dry material, most of which corresponds to phenolic acids (6.6 mg CAF/g of dry material) and flavonoids (4.7 mg QE/g).

Considering that ethanol–water is a popular solvent for tannin extraction [28], the colorimetric tannin detection method from European Pharmacopeia was applied. It has to be taken into account that in this method, the tannin content is normalized to the redox potential of pyrogallol, suggesting that there might exist biases due to different redox potentials of the different tannins (e.g., catechines vs. gallotannins) [29]. Tannins are classified as hydrolyzable (gallic acid and ellagic acid) and condensed (proanthocyanidins) tannins [30].

**Figure 2:**
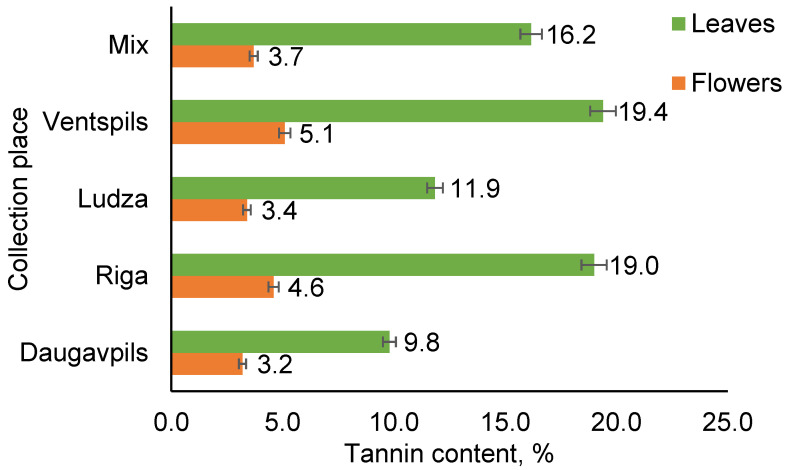
Tannin content in the leaf and flower extracts of *T. vulgare* from different collection places.

The results in Figure 2 show that the highest content of tannins is found in the extracts of leaves (10 to 19%). Relatively small amounts of tannins (3 to 5%) were observed in the flower extracts of *T. vulgare*.

It has been reported that biologically active properties of tannins not only may be attributed to tannin-rich medicinal plant extracts used in traditional medicine but also could be related to the synergy of tannins with other bioactive polyphenols present in these plants [31]. Considering that tannins affect the biological activities of extracts, it is important to monitor tannin content in *T. vulgare*.

### 2.3. Identification of Individual Phenolic Compounds in Extracts of T. vulgare

The chemical composition analysis of the leaf and flower extracts of *T. vulgare* was performed using liquid chromatography. Since the focus was on phenolic compounds, the liquid chromatography analysis was performed at the absorption maximum of these compounds at 280 nm.

**Figure 3:**
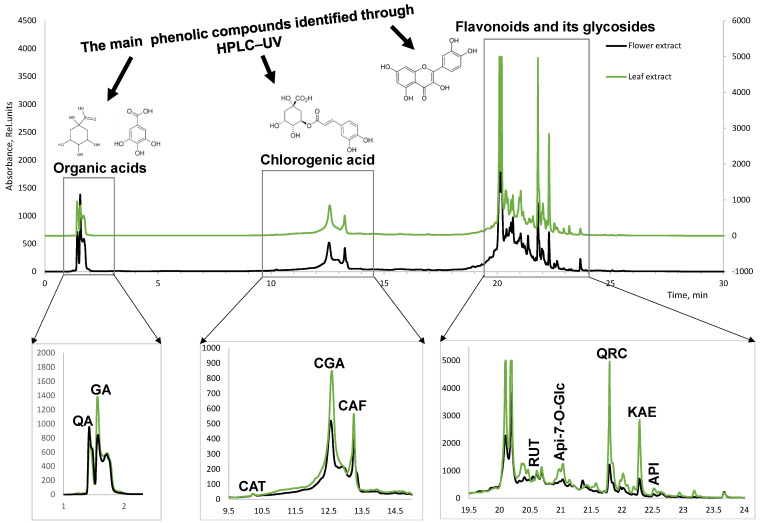
LC chromatogram of *T. vulgare* leaf and flower extracts at 280 nm. QA-quinic acid, GA-gallic acid, CAT-catechin, CGA-chlorogenic acid, CAF-caffeic acid, RUT-rutin, Api-7-O-Glc-apingenin-7-O-glucoside, QRC-quercetin, KAE-kaempherol, API-apingenin.

The identification of individual compounds (Figure 3) was carried out using ten different standard substances: quinic acid, gallic acid, chlorogenic acid, caffeic acid, catechin, quercetin, kaempferol, rutin, apingenin, and apingenin glycoside.

The identification of the abovementioned value compounds was performed by a standard addition method. The results obtained show further perspectives of *T. vulgare* wild-growing in Latvia as a potential source of these phenolic compounds.

The HPLC method provides distinctive chemical profiles, also known as fingerprints [32]. The significant difference observed was the quantitative amount of compounds in the fingerprint patterns of the leaf and flower extracts.

### 2.4. Thujone Content in Extracts of T. vulgare

As previously mentioned, the use of *T. vulgare* is limited due to the presence of thujones.

The thujone (α + β) content in the *T. vulgare* samples was less than 1% on average, but two regions, Daugavpils and Ludza, had more than 4% (Table 4). It is worth noting that these regions are geographically closer to each other than the other regions that were studied. The results suggest that in these two regions, another chemotype with different first major constituents could be more common.

The amount of thujone also varied in a wide range of extractives from the *T. vulgare* plants investigated before. The variation in the different chemical markers (camphor, thujone, and 1,8—cineole) of the essential oil in *T. vulgare* plants collected in the Baltic States has been reported [33].

### 2.5. Antioxidant Activity

The antioxidant properties of plants have become one of the most important indicators for evaluating their potential use in medicine [34]. The DPPH radical scavenging assay is the most common method used in the study of the antioxidant activity of plant extracts. The antioxidant activity of all extracts measured by the ability to scavenge (DPPH) free radicals was compared to the standard antioxidant trolox (water-soluble analogue of vitamin E).

**Figure 4:**
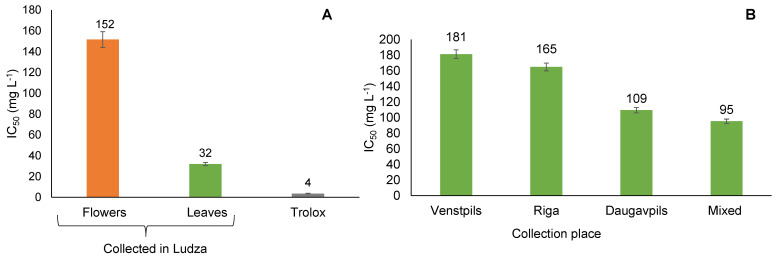
Radical scavenging activity of leaf and flower extracts (**A**) and of leaf extracts (**B**) of *T. vulgare* harvested from different collection places by DPPH test.

All extracts of *T. vulgare* were found to have concentration-dependent inhibitory activity against the DPPH radical. In this study, the crude extracts showed DPPH radical scavenging effects with IC_50_ values in the range of 32 to 181 mg/L (Figure 4). The lower the IC_50_ value, the more powerful the antioxidant capacity.

Our results suggest that the aqueous ethanol extracts of *T. vulgare* have antioxidant activity on non-biological radical DPPH but are less efficient than Trolox (3.6 ± 0.2 mg L^−1^ (n = 3)). There are four categories of antioxidant activity: very strong (IC_50_ < 50 mg L^−1^), strong (IC_50_ between 50 to 100 mg L^−1^), moderate (IC_50_ between 100 to 150 mg L^−1^), and weak (IC_50_ between 150 to 200 mg L^−1^) [35]. In our study, the extracts obtained from *T. vulgare* leaves harvested in Ludza had very strong antioxidant activity (IC_50_ = 32.4 mg L^−1^, respectively). The leaf extracts obtained from *T. vulgare* harvested in Daugavpils and the mixed leaf sample belong to the active antioxidant category because they have IC_50_ values of 109.3 mg L^−1^ and 95.2 mg L^−1^, respectively. The other aqueous ethanol extracts of *T. vulgare* showed weak antioxidant activity, with IC_50_ values between 152 to 181 mg L^−1^. The data provided in the literature shows that the crude tansy extract could have DPPH radical scavenging effects with an IC_50_ value of 37 mg L^−1^ [16]. The findings are in good agreement with this value.

In addition, the total phenolic, tannin, and flavonoid contents of the leaf extract of *T. vulgare* collected from Ludza, which has high antioxidant activity compared to other extracts, were found to be high. Therefore, these compounds can be considered responsible for the antioxidant activity of the extract. The previously reported correlation between total phenolic compounds and various in vitro tests suggests that phenolic compounds are the main carriers of antioxidant power in the extracts of *T. vulgare* [36].

## 3. Discussion

In the present study, different morphological parts (leaves, flowers, stems, and aerial parts) of *T. vulgare* were analyzed for the chemical profile of phenolic compounds and antioxidant activity. Additionally, flowers and leaves were collected from four different regions of Latvia. Moreover, along with the analysis of phenolic compounds, the potentially toxic co-eluted thujone was qualitatively and quantitatively detected.

The *T. vulgare* is a common plant in Latvia. It is widely distributed throughout the whole country as the only *Tanacetum* genus species; however, its practical application has not yet been extensively studied. Our research is the starting point for further investigations aimed at the utilization potential of *T. vulgare* growing in Latvia.

The investigations of *T. vulgare* focused mainly on volatile compounds, especially in essential oil [1,13,18,37]. However, the average amount of essential oil found in the aerial parts of *T. vulgare* plants is from 0.1% up to 1.9% [38]. Some air-dried *T. vulgare* plants have been reported to contain up to 3% essential oil [39,40]. The phenolic compound-rich extracts of *T. vulgare* can reach up to 30% [41]. It should be considered that some components of essential oil could be co-extracted with hydrophilic extractives. The essential oil of *T. vulgare* is essentially composed of terpenoids and phenolic compounds. For the separation of terpenes (the main part of essential oil) from phenolic compounds, several techniques could be applied, e.g., solid phase extraction, the Craig-type apparatus, and circular chromatography. It is known that the phytochemical profiles and the bioactivities of *T. vulgare* are heavily impacted by the type and ratio of solvents used for extraction. The extraction method depends on the chemistry of the substance to be extracted, and for efficient extraction of phenolic compounds, combinations of aqueous and organic solvents are preferred [9]. Therefore, 50% aqueous ethanol (*v*/*v*) was selected as the extraction solvent for phenolic compounds.

By comparing the experimentally obtained extraction yields of hydrophilic extractives to the data provided in the literature, the results obtained in this research show similarity towards classical extraction methods (15 to 20%, *w*/*w* [38,41]), and the numbers are relatively comparable to advanced extraction techniques applied for *T. vulgare* (20 to 30%, *w*/*w* [41]). This means that the experimentally obtained extraction yields could be further improved by advanced extraction techniques (ultrasonic-assisted extraction, accelerated solvent extraction, etc.).

*Tanacetum* populations show high variability with regard to the essential oil composition, and more than 15 distinct chemotypes have been described in Scandinavia and the Baltic countries so far [42]. The variation of secondary metabolites, depending on chemotypes, has also been reported in other natural populations [43]. While there are differences in the essential oil composition, significant variation in the content of phenolic compounds has also been detected [44]. The content of lipophilic extractives depends on the aerial part. It is generally considered that the essential oil content in the inflorescences is higher than in other aerial parts [1]. Our results show that the hydrophilic extractives in *T. vulgare* have the same tendency, and the leaves have a higher content of hydrophilic extractives than the other parts.

In a study [18], the *T. vulgare* was demonstrated to be an under-investigated plant, even though comprehensive data on its properties and chemical composition as well as processing technologies may lead to the development of valuable products for various applications. The significant need for experimentally proven scientific investigations of *T. vulgare* was emphasized by several other researchers [6,11,38]. Our research supports the knowledge-based application potential of *T. vulgare* by offering additional, alternative resources on *T. vulgare* plants in the Baltic States.

The investigation of phenolic compounds in *T. vulgare* has recently become relevant [6,38], but the available data are limited and controversial in terms of amount (84–142 GAE mg/g in Serbia and Lithuania [6,15]; 40–50 GAE mg/g in Poland and Rumania [18,26]). In addition, the groups of dominant compounds (flavonoids, organic acids, and tannins) differ significantly. The crude aqueous ethanolic extract from aerial parts of *T. vulgare* contains flavonoids, phenolic acids, coumarins, and tannins [45]. However, the concentration of phenolic compounds in *T. vulgare* depends on the type of habitat. The distribution of phenolic compounds in plants is difficult to explain by geographical differences in locations [46].

According to the data provided in the literature, depending on the harvesting region, *T. vulgare* can contain total tannins from 4.3% up to 8.75%. Based on the study about tannin content in the extracts of *T. vulgare*, it is recommended that the extracts contain no less than 4% tannins [47]. However, only limited information is available on the chemical characterization of *T. vulgare* tannins. It has been reported that in the inflorescences part and leaves, hydrolyzable tannins are dominant [48]. The tannin content is of high importance because the topicality of tannin application in various industries (pharmaceutical, cosmetic, food, etc.) grows very fast [49]. Plant tannins are more abundant in vulnerable parts of plants, such as new leaves and flowers. The presence of tannins is highly variable among different plants, growth stages, and morphological parts [50].

Quinic acid, caffeic acid, and their esterified derivatives have been reported as characteristic non-volatile compounds in *T. vulgare* [38]. Gallic acid has been mentioned as one of the biologically active constituents of the liquid extract (ethanol 70%) of the common tansy herb recommended as a hepatoprotective agent [51]. All flavonoids (catechin, quercetin, kaempferol, rutin, and apingenin) identified in our research have been previously reported in *T. vulgare* as value components with a significant contribution to pharmaceutical activities [6,47,52]. Among the metabolites identified in *T. vulgare*, phenolic compounds, including caffeoylquinic acids and flavonoids, were considered a particularly important group because they contribute to the major multifunctional biological activity that may be linked to their antioxidant potential [53]. The performed chemical analyses allowed us to highlight the differences in the profiles of the leaves and flowers as well as to evaluate the variability within each biomass of *T. vulgare* depending on the harvesting location. It was found that the leaves and inflorescences of the *T. vulgare* plant synthesize the same phenolic compounds but of different compositions. The comparable results of the chemical composition and antioxidant activity detected in our study fit well with already reported findings of *T. vulgare* plants harvested in other regions, supporting the evidence-based opportunities of practical application.

The significant finding of our study was the co-eluted thujone in ethanol–water extracts. Considering that *T. vulgare* is one of the plant species that accumulates thujones [19], it is important to detect the amount of these constituents. Thujone is a volatile monoterpene ketone that is usually present in nature in two stereoisomers, α-thujone and β-thujone. For regulatory purposes, the sum of both isomers is generally assessed [54].

More than 15 different chemotypes of *T. vulgare* from Scandinavia and the Baltic states have been described, and most researchers identify β-thujone, α-thujone, camphor, and chrysanthenyl acetate as the main components of the essential oil [1]. Thujone occurs in different quantities in *Tanacetum* species [19]. The widespread nature of the thujone-chemotype essential oil has been reported in 15 countries on the European and American continents [33].

Thujones are generally characterized by gas chromatography methods [55]. Considering that the predicted content of thujone in the analyzed hydrophilic extracts must be low, the extract solubility in organic solvents could be lower as well. Therefore, the liquid chromatography method is more suitable for thujones characterization in hydrophilic extracts [56,57].

Although thujones have toxic effects, estimates for the allowable daily intake via herbal preparations and diet for humans are 3 to 7 mg/day [58]. Furthermore, the European Medicines Agency (EMA) also proposed a daily maximum intake of thujone in *Absinthii herba*, which was established at 3.0 mg thujone/day/person as acceptable for a maximum duration of 2 weeks in the wormwood (*A. absinthium*) monograph. Low doses of thujone may activate the DNA repair mechanism and act as antigenotoxic agents [19]. There are still important gaps in the knowledge required to assess thujone toxicity, the most important being human dose–concentration–effect relationships, including the elucidation of bioavailability and the actual toxicological consequences of potential pharmacogenetic variations [58]. Thujone-containing species are frequently used as natural remedies in ethnobotanical applications. However, it has been demonstrated that global climate change often alters the chemical composition of plants [59]. The potentially toxic compounds must be constantly monitored in plants.

The extracts obtained from *T. vulgare* leaves have not been reported to have significant toxicity. In view of the dose of *T. vulgare* consumed in traditional medicine, there is a wide margin of safety for the therapeutic use of the extracts of *T. vulgare* leaves [60].

According to the information available in the literature, thujone (α + β) has previously been reported only for the essential oil of *T. vulgare*.

The antioxidant activity found in the extract is supported by the results reported in other studies of *T. vulgare* and the traditional medicinal uses of the plant for various conditions such as injury recovery, arthritis, inflammatory processes, etc. [45].

## 4. Materials and Methods

### 4.1. Plant Material

The study object was wild-growing tansy plants (*Tanacetum vulgare* L.) in Latvia. The aerial parts (flowering tops up to lengths of 20 cm, flowers, leaves, and stems) of the wild tansy plants were harvested in the period of mass flowering (August 2022) from four different localities in Latvia: Ventspils (57°14′40″ N 21°39′44″ E), Riga (56°54′18″ N 24°05′57″ E), Daugavpils (55°53′40″ N 26°16′35″ E), and Ludza (56°32′32″ N 27°43′20″ E). The plants were identified by Prof. Dace Bandere, Ph.D. in Pharmacy. The voucher herbariums of the plants are kept in the internal collection of Riga Stradiņš University Department of Pharmaceutical Chemistry and labeled BLM-2022; BLV-2022; BLL-2022; BLR-2022; BLD-2022; BZM-2022; BZV-2022; BZL-2022; BZR-2022; and BZD-2022. The plants were dried at room temperature (20 to 25 °C) in shade. All the samples were dried to equilibrium moisture content and ground in a mill to obtain particles with a size of <2 mm.

### 4.2. Chemicals and Reagents

Reference standards: caffeic acid (≥98%), quinic acid (≥98%), catechin (≥98%), apigenin (≥95%), and apigenin-7-glucoside were purchased from Sigma-Aldrich (St. Louis, MO, USA). Rutin was purchased from PhytoLab (Vestenbergsgreuth, Germany), and chlorogenic acid was purchased from the HWI group (Rülzheim, Germany). All solvents used were analytical or HPLC grade. The water was distilled and purified using the Stakpure GmpH water system (Niederahr, Germany). Gallic acid and AlCl_3_ and Trolox were purchased from Acros Organics (Geel, Belgium), and Na_2_CO_3_ and NaNO_2_ were obtained from Honeywell (Charlotte, NC, USA). 2,2-diphenyl-1-picrylhydrazyl (DPPH) was from Alfa Aesar (Kandel, Germany). Folin–Ciocalteu reagent, H_2_SO_4_, HCl, and NaOH reagents were purchased from Fisher Scientific (Loughborough, UK). LC-grade acetonitrile, methanol, and formic acid were purchased from Sigma-Aldrich (St. Louis, MO, USA), and water for LC analysis was purified using a Stakpure GmpH water purification system (Niederahr, Germany).

### 4.3. Moisture Content

The gravimetric method was applied for the determination of the moisture content. The weight loss on drying was measured by an oven method, dried at 105 °C for 3 h. The 5 g tansy sample was accurately weighed and dried to a constant mass in a vacuum oven at 105 °C for 3 h. The sample was reweighed after 30 min, and the moisture content was calculated and expressed as the percentage moisture content of the fresh weight.

### 4.4. Extraction

The 5.00 g of air-dried fine tansy powder was extracted with 50 mL of 50% aqueous ethanol by maceration with shaking using an orbital shaker (PSU-10i Biosan, Riga, Latvia) for 24 h at room temperature. Subsequently, the mixture was filtered through a 9 mm diameter filter paper (Sartorius, FT-3-303-110, Goettingen, Germany). The ethanol was evaporated in a rotary vacuum evaporator (Heidolph Laborota 4002 control, Schwabach, Germany), and extracts in water were freeze-dried by lyophilization at −80 °C, 0.05 mBar.

### 4.5. Total Phenolic Content

The total phenolic content was determined using the Folin–Ciocalteu method with minor modifications using gallic acid as the standard [27]. A total of 5 to 10 mg of the lyophilized extracts were dissolved in 25 mL of 50% ethanol. Briefly, 400 µL of extracts were mixed with 4 mL of 7.5% sodium carbonate and 5 mL of 10% Folin–Ciocalteu phenol reagent, and then the mixture was vortexed briefly. After incubation for 30 min at room temperature in the dark, absorbance was read at 750 nm using an ultraviolet (UV)-visible spectrophotometer (Mettler Toledo, LabX™, Greifensee, Switzerland). Total phenolic content was calculated based on a calibration curve of gallic acid. The results were expressed as milligram gallic acid equivalents per gram of lyophilized extract and tansy plant (milligram GAE per gram dry weight (DW)).

### 4.6. Total Flavonoid Content

The total flavonoid content was determined according to Sun et al. [61] with slight modifications as follows. A total of 5 to 10 mg of the lyophilized extracts were dissolved in 25 mL of 96% ethanol. Then, 0.4 mL of the extract solutions was added to a 10 mL test tube containing 2 mL of water. After that, 0.12 mL of 5% sodium nitrite (NaNO_2_) solution was added and incubated for 5 min at room temperature, and then 0.24 mL of 10% aluminum nitrate solution was added to the mixture. After 6 min, 0.8 mL of 1 mol/L sodium hydroxide was added. The absorbance was measured at 420 nm. The equation obtained for the calibration curve of the standard quercetin solution was y = 1.0369x − 0.0228 (R^2^ = 0.9954), and the result was expressed as mg QE/g (quercetin equivalents) per gram of dry weight (DW) extract.

### 4.7. Total Phenolic Acids

The method determined the total content of phenolic acids based on the proportional increase in the color intensity of the solution relating to the content of phenolic acids in the test [1]. A total of 10 to 15 mg of the lyophilized extracts were dissolved in 10 mL of water. The test solution was prepared by measuring 1 mL of extract solution, 1 mL of distilled water, 1 mL of 0.5 M hydrochloric acid, and 1 mL of Arnov’s reagent (10 g of sodium molybdate and 10 g of sodium nitrite dissolved in water and supplemented to 100 mL) into measuring tubes with a capacity of 10 mL. After 6 min, 1 mL of 0.1 M sodium hydroxide was added, supplemented with 5 mL of distilled water. The absorbance was measured at 490 nm. The total contents of phenolic acids in terms of caffeic acid were calculated according to the following formula, y = 0.7505x − 0.0505 (R^2^ = 0.9993), and the result was expressed as mg CAF/g (caffeic acid equivalents) per gram of dry weight (DW) extract.

### 4.8. Total Tannin Content

The analyses of the extracts were carried out in accordance with the monograph 2.8.14 available in the European Pharmacopoeia. In short, according to Neumann 2022 [29], the drugs were pulverized, and approximately 500 mg was extracted with boiling water (150 mL) for 30 min. The water extract was filtered and split into two parts of equal volume. Half of the filtered water extract was mixed with hide powder CRS and filtered once again. Phosphomolybdotungstic reagent R was added to both filtered water extracts, and their pH was adjusted using a sodium carbonate solution. The solutions were allowed to equilibrate for approximately 30 min. The total polyphenol contents were then obtained by measuring the absorbance of the solutions at a wavelength of 760 nm. Pyrogallol mixed with phosphomolybdotungstic reagent R was used as the standard. Since the hide powder CRS adsorbs the tannin polyphenols, it was possible to quantify the total tannin content by calculating the concentration difference between the two results obtained from the absorbance measurements. The hide powder CRS is a reference standard of the European Pharmacopoeia for the detection of phenolic compounds and tannins. The tannin content was calculated in the dried extracts.

### 4.9. Identification of Individual Phenolic Compounds

Standard stock solutions of ten different substances—quinic acid, gallic acid, chlorogenic acid, caffeic acid, catechin, quercetin, kaempferol, rutin, apingenin, and apingenin glycoside—were prepared in 50% acetonitrile (2 mg/mL). All solutions were filtered prior to analysis through a 0.45 μm syringe filter and injected three times into the HPLC. The phenolic compounds were identified by analyzing the same extracts with no added standard compounds and with added standard compounds. LC analysis was performed on a DIONEX UltiMate 3000 UHPLC+ focused system (Thermo Fisher Scientific, Waltham, MA, USA), which consists of an UltiMate 3000 pump, UltiMate 3000 autosampler, UltiMate 3000 column compartment, UltiMate 3000 variable wavelength detector, and Chromeleon software. As a stationary phase, an Ascentis Express 90 Å AQ-C18 column (15 cm × 3.0 mm, 2.7 μm, Supelco, Darmstadt, Germany) was used. The mobile phase was composed of 0.1% aqueous trichloroacetic acid (*v*/*v*) (A) and acetonitrile (B) with the following gradient elution: 0 min—95% A, 10 min—75% A, 25 min—20% A, and 30 min—95% A. Before each sample analysis, a 4 min equilibration with 5% B was performed. The flow rate was set at 0.425 mL/min, the column temperature was 40 °C, the injection volume was 1 μL, and the time of analysis was 30 min.

### 4.10. Determination of Thujone Content

The purified standard of α, β-thujone (80% purity; 70% α-thujone and 10% β-thujone) was from Sigma-Aldrich (Steinheim, Germany). The reference standard solution contained 0.724 g/mL of α- and β-thujone (taking into consideration a purity of 80.0% and density of 0.925 g/mL). An aliquot of 25 μL of the standard solution was diluted in 25 mL of methanol to a final concentration of 0.724 mg/mL. The extracts were dissolved in methanol with an approximate of concentration 2 to 6 mg/mL, filtered (Nylon filter, 0.45 μm pore size), and then used for reversed phase liquid chromatography (DIONEX UltiMate 3000 UHPLC, Thermo Fisher Scientific, Waltham, MA, USA) with fluorescence detection experiments. The peak areas were recorded, and the amount of thujone was calculated using the calibration plot on LC.

The LC analysis was carried out using a Zorbax Eclipse XDB-C18 (Agilent, 5 µm, 15 cm × 0.46 cm i.d.) column heated at 35 °C and an acetonitrile to water ratio of 55 to 45 as the mobile phase; the flow rate was 1 mL/min, and the injection volume was 20 µL. The fluorescence detector was set at 220/290 nm (λex/λem). The thujones were detected as only one peak as the sum of the two isomers. The quantitative analysis was performed both by using linear regression analysis, obtained by injecting various concentrations of thujone solution in methanol, and by the standard addition method.

### 4.11. Antioxidant Activity Using DPPH Method

The antioxidant capacity was determined with the 2,2-diphenyl-1-picrylhydrazyl (DPPH) radical scavenging assay [62]. The 10 µg/mL DPPH solution was prepared in methanol. The antioxidant activities were expressed as the half maximal inhibitory concentration (IC_50_), defined as the antioxidant concentration needed to reduce the absorbance of the control by 50%. The lower the IC_50_ value, the higher the antioxidant activity. A sample solution in DMSO (0.03 mL) was mixed for 15 min with 3.0 mL of a DPPH in methanol solution, and then the absorbance at 517 nm of the mixture was immediately measured using a Metter Toledo UV7 spectrometer.

### 4.12. Statistical Analysis

The measured values were shown as an average with a confidence interval (at a level of significance α = 0.05). Each measurement was performed at least in triplicate. Statistical calculations were carried out using Microsoft Office Professional Plus 2019 Excel.

## 5. Conclusions

In terms of the extraction efficiency of hydrophilic extractives (up to 20% *w*/*w* for leaves and up to 16% *w*/*w* for flowers), the flowers and leaves of *T. vulgare* have been proven to be promising for the development of new herbal products.

Phytochemical screening of the ethanol–water extracts of *T. vulgare* showed phenolic compounds, mainly tannins, flavonoids, and phenolic acids. The total tannin content in tansy leaf extracts was up to 19%, and considering its potential biological activities and synergic effect with other compounds, tannins should be characterized in more detail. The value constituents were identified as chlorogenic acid, apigenin and its glycoside, quinic acid, rutin, and kaempherol.

The chemical profile of *T. vulgare* leaves and flowers was dependent on the sampling location. Thujone variation was significantly high not only between aerial parts (leaves, flowers) but also between collection sites. The toxic thujone content should be controlled in all extracts of *T. vulgare* regardless of the plant part or extraction solvent used. The aqueous ethanol extracts of *T. vulgare* have antioxidant activity (IC_50_ between 181 to 32 mg L^−1^) on non-biological radical DPPH but are less efficient than Trolox, a hydrophilic analogue of vitamin E (IC_50_ 3.6 ± 0.2 mg L^−1^).

Further studies may obtain data on important relationships between the chemical composition and biological activities of *T. vulgare* extracts.

The data obtained from this study are indicative and may be helpful for future cultivation and the practical application of *T. vulgare* plants.

## Figures and Tables

**Table 1:** Extraction yield of different morphological parts of wild *T. vulgare* harvested in Daugavpils, *w*/*w* (%).

Morphological Part	Extraction Yield *, %
Leaf	18.0 ± 0.2
Flower	8.2 ± 0.1
Stem	10.9 ± 0.1
Aerial part	14.3 ± 0.3

* Data are expressed as mean ± SD (n = 3).

**Table 2:** Total phenolic, flavonoid, and phenolic acid content in leaf and flower extracts of *T. vulgare* collected in different places.

Sample	Total Phenols *, mg GAE/g		Total Flavonoids *, mg QE/g		Total Phenolic Acids *, mg CAF/g	
	Flower	Leaf	Flower	Leaf	Flower	Leaf
Mix	137.67 ± 4.21	146.25 ± 3.98	17.60 ± 0.55	24.79 ± 0.48	12.49 ± 0.61	21.07 ± 0.64
Ventspils	126.90 ± 3.45	135.17 ± 3.70	14.58 ± 0.51	26.68 ± 0.53	10.98 ± 0.53	19.96 ± 0.46
Ludza	155.38 ± 4.71	218.87 ± 4.09	19.53 ± 0.49	27.60 ± 0.56	16.85 ± 0.56	29.31 ± 0.39
Riga	138.05 ± 3.19	147.44 ± 3.21	15.27 ± 0.43	25.17 ± 0.49	14.44 ± 0.41	30.46 ± 0.61
Daugavpils	134.20 ± 4.16	155.46 ± 3.96	16.20 ± 0.51	25.89 ± 0.38	15.15 ± 0.45	31.21 ± 0.54

* Data are expressed as mean ± SD (n = 3). GAE—gallic acid equivalents; QE—quercetin equivalents; CAF—caffeic acid equivalents.

**Table 3:** Total phenolic, flavonoid, and phenolic acid content in extracts obtained from different morphological parts of *T. vulgare ^§^*.

Morphological Part	Total Phenols *, mg GAE/g		Total Flavonoids *, mg QE/g		Total Phenolic Acids *, mg CAF/g	
	Extract	*T. vulgare*	Extract	*T. vulgare*	Extract	*T. vulgare*
Leaves	155.46 ± 3.51	27.98 ± 0.42	25.89 ± 0.49	4.66 ± 0.41	31.21 ± 0.48	6.58 ± 0.41
Flowers	134.20 ± 2.37	11.04 ± 0.28	16.20 ± 0.42	1.33 ± 0.33	15.15 ± 0.36	2.53 ± 0.32
Stems	127.35 ± 1.52	15.91 ± 0.41	12.85 ± 0.33	2.15 ± 0.40	19.54 ± 0.52	2.44 ± 0.39
Aerial part	239.84 ± 3.36	24.61 ± 0.42	84.53 ± 0.81	8.67 ± 0.19	22.27 ± 0.48	2.28 ± 0.18

*^§^* Harvested in Daugavpils. * Data are expressed as mean ± SD (n = 3).

**Table 4:** Thujone content (%) in leaf and flower extracts of *T. vulgare*.

Sample	Thujone Content *, %	
	Flowers	Leaves
Mix	0.82 ± 0.02	0.48 ± 0.02
Ventspils	0.67 ± 0.01	0.63 ± 0.04
Ludza	5.98 ± 0.12	5.44 ± 0.16
Riga	0.91 ± 0.02	0.44 ± 0.02
Daugavpils	4.49 ± 0.19	3.82 ± 0.09

* Data are expressed as mean ± SD (n = 3).

## Data Availability

The data presented in this study are available on request from the corresponding authors.
